# Factors associated with predictors of smoking cessation from a Norwegian internet-based smoking cessation intervention study

**DOI:** 10.18332/tpc/155287

**Published:** 2022-10-31

**Authors:** Inger T. Gram, Konstantinos Antypas, Silje C. Wangberg, Maja-Lisa Løchen, Dillys Larbi

**Affiliations:** 1 Department of Community Medicine, Faculty of Health Sciences, UiT The Arctic University of Norway, Tromsø, Norway; 2 Norwegian Centre for eHealth Research, University Hospital of North Norway, Tromsø, Norway; 3 SINTEF Digital, Oslo, Norway; 4 Department of Health and Care Sciences, UiT The Arctic University of Norway, Tromsø, Norway

**Keywords:** prevention, smoking, tobacco control, global health, predictors of smoking cessation, snus use

## Abstract

**INTRODUCTION:**

We examined if we could identify predictors for smoking cessation at six months post cessation, among smokers enrolled in a large Norwegian population-based intervention study.

**METHODS:**

We followed 4333 (72.1% women) smokers who enrolled in an internet-based smoking cessation intervention during 2010–2012. The baseline questionnaire collected information on sociodemographic and lifestyle factors, including current snus use. The cessation outcome was self-reported no smoking past seven days, at six months. We used logistic regression to estimate odds ratios (ORs) with 95% confidence intervals, to identify predictors of smoking cessation, adjusting for potential confounders.

**RESULTS:**

Women (OR=1.30; 95% CI: 1.01–1.69) compared with men, and those with medium (OR=1.31; 95% CI: 1.02–1.68) and longer (OR=1.42; 95% CI: 1.06–1.90) education compared with those with shorter education, were more likely to be successful quitters.

Overall, being a student (OR=0.56; 95% CI: 0.37–0.85) compared with having full-time work, and a moderate to high Fagerström test for nicotine dependence (FTND) score (OR=0.69; 95% CI: 0.55–0.87) compared with a low score, were predictors for unsuccessful cessation. Current snus use was a predictor for unsuccessful cessation compared to no snus use for both men (OR=0.49; 95% CI: 0.28–0.88) and women (OR=0.49; 95% CI: 0.32–0.75).

**CONCLUSIONS:**

Our study identifies female sex and longer education as predictors for successful smoking cessation, while a medium or high FTND score, being a student, and current snus use, were predictors for unsuccessful smoking cessation. Only current snus use was a predictor for unsuccessful cessation for both sexes. Our results indicate that smokers should be warned that snus use may prevent successful smoking cessation.

## INTRODUCTION

Smoking is a leading cause of preventable death^1^. Promoting reduced tobacco use is a simple and cost-effective measure to reduce premature death and disability^2^. In Norway, the prevalence of daily smoking peaked at 65% during the late 1950s for men and at 37% in 1970 for women. The noticeable decrease started during the early 1970s for men and at the turn of the millennium for women^3^. During the last two decades, the prevalence of smoking has continued to decrease, while that of snus, a moist oral tobacco product placed under the upper lip, has increased, especially amongst younger women^4^.

In a report, based on data from seven cross-sectional Norwegian surveys, Lund et al.^5^ suggested, based on the inverse correlation of smoking and snus prevalence, that snus use may help smokers to quit smoking. In 2019, Clarke et al.^6^ in their review claimed that the low smoking prevalence in Norway was due to the increase in snus use.

There is limited information on the association between the use of snus and smoking cessation and other factors associated with smoking cessation in Norway. The ongoing debate has, to a large extent, been based on results from cross-sectional studies. It is necessary to have data from follow-up studies to be able to say something about predictors and outcome.

In 2021, Cheung et al.^7^ noted that there is a lack of knowledge from randomized controlled trials (RCTs) and follow-up studies regarding factors that are related to smoking cessation at a population level.

We have previously conducted one of the largest internet-based smoking cessation RCTs, with more than 4000 enrolled smokers with a six-month follow-up period post cessation. Most of the smoking cessation RCTs have shorter follow-up periods. Compared with RCTs with a similar follow-up period, we had a relatively high quit rate, as one in nine reported to be smoke-free at six months post cessation. This was in both arms, regardless of delivery methods. Consequently, we decided to do a secondary analysis of these data^8^.

The aim of this study was to examine if we could identify predictors for smoking cessation at six months post cessation, among smokers enrolled in a large Norwegian population-based intervention study.

## METHODS

### Data source

The study participants were smokers who had enrolled in an open, free, Norwegian internet-based, smoking cessation program^9^. The study was approved by the Regional Committee for Medical and Health Research Ethics. The participants gave their informed consent when they registered on the slutta.no (‘stop. no’) website. The study found that smoking cessation interventions delivered by mobile text messaging and email may be equally successful at the population level. At six months follow-up, in both arms, approximately one in five responded. Furthermore, in both arms, one in nine enrolled smokers reported 7-day point prevalence abstinence, six months post cessation. Details of the randomized double-blinded control trial, which was conducted during 2010–2012, are described elsewhere^8^.

### Measures

*Baseline variables*

The baseline questionnaire collected information on sex, age, duration of education in years, married/living with a partner (yes, no), occupational status (full-time work, part-time work or homemaker, welfare recipients, students, and unemployed). The questionnaire asked, among other things: about the number of quit attempts; current snus [moist tobacco (snuff)] use (none, occasionally, daily); cohabitant smoking (yes, no); and friends smoking (none/few, many, all). The questionnaire included questions related to the FTND: number of cigarettes smoked per day (≤10, 11–20, 21–30, ≥31); ‘do you smoke in the morning (yes, no)?’; ‘is the first cigarette smoked in the morning the best (yes, no)?’; the time between waking up and smoking (≤5, 6–30, 31–60, >60 min); ‘do you smoke in places where it is prohibited (yes, no)?; and ‘do you smoke when ill or bedridden (yes, no)?’. From these, we could calculate FTND score (0–10 points) and classify nicotine dependence according to the categories: low (≤3 points); medium (4–6 points); and high (≥7 points)^10^. The age variable was for a short period incorrectly recorded at enrollment due to a technical error. Age as an inclusion criterion was not affected.

*Smoking cessation outcome*

At six months post cessation, participants who answered ‘no’ to both of the following questions: ‘Are you currently smoking?’ and ‘Have you been smoking, even as little as one single puff during the past 7 days?’ were counted as successful quitters. We used an intention to treat analysis for the outcome. This means that all remaining participants, who had completed the six months’ time-point, including those lost to follow-up (n=3580), were counted as not having quit smoking. The response rate, and thus the attrition rate, did not vary by study arm at six months post cessation^8^.

### Statistical analysis

We calculated percentages (%), means with standard deviation (± SD), or median with interquartile range, for the distribution of selected characteristics at baseline, overall, and by sex. We used t-tests and Pearson’s chi-squared tests to analyze the differences between men and women.

We used logistic regression models to estimate crude and multivariable adjusted odds ratios with 95% confidence intervals to identify predictors of successful and unsuccessful smoking cessation at six months. We calculated the attrition rate (%) for men, women, current snus users, and non-snus users at six months post cessation. Including only responders, we used logistic regression models to estimate multivariable adjusted odds ratios to compare successful and unsuccessful smoking cessation at six months for women versus men, and snus users versus non-snus users. We used the Statistical Package for the Social Sciences for Windows, the 25th version (IBM SPSS 25.0) for all analyses. We considered two-sided p<0.05 as significant.

## RESULTS

In total, 4333 (72.1% women) smokers enrolled in the study. Table 1 shows that at enrollment, the mean age was 39 years, the majority of participants (59.4%) had ≥13 years of education, 3 in 4 (76.8 %) had a cohabitant, and most of the participants (55.9%) worked full-time.

**Table 1:** Distribution (%)^a^ of selected characteristics at enrolment, overall and by sex among participants enrolled in an internet- and mobile-based smoking cessation intervention in Norway, 2010–2012 (N=4333)^b^

*Characteristics*	*All (n=4333) %*	*Men (n=1210) %*	*Women (n=3123) %*	*p^c^*
**Age** (years), mean (SD)	39.39 (11.37)	40.53 (11.69)	38.92 (11.22)	0.008
**Education duration** (years), n	4320	1207	3113	
≤12	40.6	44.5	39.1	0.000
13–16	39.4	34.4	41.4	
≥17	20.0	21.1	19.5	
**Cohabitants**, n	4333	1210	3123	
Yes	76.8	73.3	78.2	0.001
**Cohabitant smoking**, n	4314	1204	3110	
Yes	33.1	32.7	33.2	0.774
**Friends smoking**, n	4333	1210	3123	
None^d^/a few	40.2	42.6	39.3	0.130
Many	56.9	54.9	57.7	
All	2.9	2.6	3.0	
**Occupational status**, n	4333	1210	3123	
Full-time	55.9	69.3	50.7	0.000
Part-time/homemaker	14.1	4.5	17.9	
Welfare recipient	13.0	11.1	13.8	
Student	11.3	9.5	12.0	
Unemployed	5.7	5.7	5.6	
**Age started smoking**, mean (SD)	15.84 (3.62)	15.98 (3.87)	15.78 (3.52)	0.097
**Number of cigarettes**, mean (SD)	15.78 (6.68)	17.19 (7.32)	15.23 (6.34)	0.000
**Quit attempts**, n	4333	1210	3123	
Never	15.0	15.6	14.7	0.432
Once	16.8	17.9	16.4	
2–3 times	37.8	36.2	38.4	
≥4 times	30.4	30.3	30.5	
**Number of quit attempts**, mean (SD)^e^	4 (7)	5 (9)	4 (6)	0.017
**Fagerström score**, n	4236	1175	3061	
Low 0–3	29.4	26.4	30.6	0.000
Medium 4–6	64.3	64.3	64.2	
High 7–10	6.3	9.3	5.2	
**Current snus use**, n	4333	1210	3123	
No	79.9	69.4	84.0	0.000
Occasionally	14.2	19.3	12.2	
Daily	5.9	11.2	3.8	

a Proportion (%) unless otherwise noted.

b Due to missing values the sum is not similar for the different variables.

c t-test or chi-squared test for difference between men and women.

d Only 33 participants had none of their friends smoking.

e Among previous quitters.

The mean age for starting smoking was 16 years, the average number of cigarettes smoked per day was 16, and the majority (85%) had tried to quit smoking with an average number of four attempts. Altogether, 64.3% gave answers that corresponded to a medium (4–6) FTND score and 6.3% to the highest (7–10) score. One in five (20%) reported being current snus users.

Compared with men, women reported being younger, more educated, having a cohabitant, not having a full-time job, and not using snus. Furthermore, women reported smoking on average fewer cigarettes, and a lower proportion had a high nicotine dependence score compared with men (all p<0.05) (Table 1).

Table 2 shows that after multivariable adjustment, women were 30% (OR=1.30; 95% CI: 1.01–1.69) more likely to achieve successful smoking cessation, compared with men. Smokers who reported duration of education to be 13–16 years were 31% (OR=1.31; 95% CI: 1.02–1.68) and those reporting ≥17 years were 42% (OR=1.42; 95% 1.06–1.90) more likely to achieve smoking cessation, compared with those reporting <13 years. Students were 44% (OR=0.56; 95% CI: 0.37–0.85) less likely to achieve smoking cessation compared with full-time workers. This association was very strong for men (OR=0.09; 95% CI: 0.01–0.69), but not for women (OR=0.70; 95% CI: 0.46–1.08). Smokers who were categorized to have a medium or high FTND score were 29% (OR=0.71; 95% CI: 0.57–0.88) less likely to achieve smoking cessation compared with those with a low score. This association was only significant for women (OR=0.68; 95% CI: 0.53–0.87).

**Table 2:** Crude (OR) and multivariable adjusted^a^ odds ratios (AOR) of smoking cessation at 6 months, overall and by sex, according to selected characteristics at enrollment in Norway, 2010–2012 (N=4333)^b^

*Characteristics*	*OR (95% CI)*	*AOR^a^ (95% CI)*		
		*Overall*	*Men*	*Women*
**Sex** (men) (Ref.)	1	1		
Women	1.42 (1.11–1.81)	1.30 (1.01–1.69)	NA	NA
**Education duration** (years)				
≤12 (Ref.)	1	1	1	1
13–16	1.43 (1.13–1.81)	1.31 (1.02–1.68)	1.77 (1.04–3.01)	1.21 (0.91–1.60)
≥17	1.59 (1.20–2.09)	1.42 (1.06–1.90)	1.75 (0.97–3.15)	1.35 (0. 96–1.89)
**Cohabitants**				
No (Ref.)	1	1	1	1
Yes	0.95 (0.75–1.21)	0.98 (0.76–1.28)	0.94 (0.55–1.60)	0.99 (0.73–1.35)
**Cohabitant smoking**				
No (Ref.)	1	1	1	1
Yes	0.77 (0.62–0.97)	0.85 (0.66–1.09)	0.74 (0.43–1.29)	0.88 (0.66–1.17)
**Friends smoking**				
No/few (Ref.)	1	1	1	1
Many/all	0.70 (0.57–0.86)	0.81 (0.65–1.00)	0.89 (0.56–1.41)	0.78 (0.61–1.00)
**Occupational status**				
Full-time (Ref.)	1	1	1	1
Part-time/homemaker	0.80 (0.59–1.09)	0.82 (0.59–1.13)	0.51 (0.12–2.18)	0.85 (0.61–1.20)
Welfare recipient	0.87 (0.64–1.19)	0.94 (0.67–1.30)	0.65 (0.29–1.46)	1. 03 (0.72–1.49)
Student	0.53 (0.36–0.79)	0.56 (0.37–0.85)	0.09 (0.01–0.69)	0.70 (0.46–1.08)
Unemployed	0.47 (0.27–0.84)	0.57 (0.32–1.02)	1.01 (0.39–2.66)	0.45 (0.21–0.93)
**Quit attempts**				
Never (Ref.)	1	1	1	1
Once	0.92 (0.65–1.32)	0.87 (0.61–1.26)	0.87 (0.45–1.72)	0.88 (0.57–1.36)
2–3 times	0.86 (0.63–1.16)	0.81 (0.59–1.12)	0.53 (0.28–1.01)	0. 95 (0.66– 1.38)
≥4 times	0.92 (0.67–1.26)	0.83 (0.60–1.15)	0.65 (0.34–1.23)	0.93 (0.63–1.36)
**Fagerström score**				
Low 0–3 (Ref.)	1	1	1	1
Medium/high 4–10	0.69 (0.55–0.85)	0.71 (0.57–0.88)	0.83 (0.51–1.36)	0.68 (0.53–0.87)
**Current snus use**				
No (Ref.)	1	1	1	1
Yes	0.46 (0.33–0.63)	0.49 (0.35–0.69)	0.49 (0.28–0.88)	0.49 (0.32–0.75)

a Adjusted for all the variables in the table were applicable.

b Lost to follow-up counted as smokers.

Those who reported being current snus users were 51% (OR=0.49; 95% CI: 0.35–0.69) less likely to achieve smoking cessation compared with those who did not. When we stratified by sex, current snus use was significantly associated with unsuccessful smoking cessation for both men (OR=0.49; 95% CI: 0.28–0.88) and women. (OR=0.49; 95% CI: 0.32–0.75) (Table 2).

Table 3 shows that for current snus users, those who had previously tried to quit 2–3 times were 79% (OR=0.21; 95% CI: 0.08–0.55) and those who had previously tried ≥4 times were 59% (OR=0.41; 95% CI: 0.17–0.95) less likely to successfully quit compared with those who had never tried. Participants were 29% less likely to have successfully quit smoking if they had a medium or high FTND score compared with low score, for both current snus (OR=0.71; 95% CI: 0.36–1.40) and non-snus (OR=0.71; 95% CI: 0.56–0.89) users. Non-snus users showed basically similar results as the overall results (Table 3).

**Table 3:** Multivariable adjusted^a^ odds ratios of smoking cessation at 6 months by current snus use, according to selected characteristics, all at enrollment in Norway, 2010–2012 (N=4333)^b^

*Characteristics*	*Current snus use*	
	*Yes (n=871) OR^a^ (95% CI)*	*No (n=3462) OR^a^ (95% CI)*
**Sex** (men) (Ref.)	1	1
Women	1.51 (0.76–2.99)	1.27 (0.96–1.67)
**Education duration** (years)		
≤12 (Ref.)	1	1
13–16	0.87 (0.39–1.94)	1.37 (1.05–1.78)
≥17	1.67 (0.73–3.79)	1.36 (0.99–1.86)
**Cohabitant smoking**		
No (Ref.)	1	1
Yes	0.90 (0.44–1.82)	0.83 (0.65–1.07)
**Friends smoking**		
No/few (Ref.)	1	1
Many/all	1.03 (0.51–2.08)	0.79 (0.63–0.99)
**Occupational status**		
Full-time (Ref.)	1	1
Part-time/home	0.38 (0.11–1.33)	0.88 (0.63–1.24)
Welfare recipient	0.65 (0.19–2.25)	0.97 (0.69–1.37)
Student	0.38 (0.14–1.04)	0.60 (0.38–0.94)
Unemployed	0.73 (0.16–3.30)	0.55 (0.29–1.04)
**Quit attempts**		
Never (Ref.)	1	1
Once	0.70 (0.29–1.72)	0.92 (0.62–1.37)
2–3 times	0.21 (0.08–0.55)	0.97 (0.69–1.36)
≥4 times	0.41 (0.17–0.95)	0.93 (0.65–1.33)
**Fagerström score**		
Low 0–3 (Ref.)	1	1
Medium or high	0.71 (0.36–1.40)	0.71 (0.56–0.89)

a Adjusted for all the variables in the table, were applicable.

b Lost to follow-up counted as smokers.

At six months post cessation, 74% of the responders were women and 12.7% were snus users. The attrition rate was for men 84.0%, for women 82.1%, for snus users 89%, and for non-snus users 81%. Compared with women, men were 12% (OR=0.88; 95% CI: 0.73–1.05) less likely, and compared with non-snus users, snus users were 47% (OR=0.53; 95% CI: 0.42–0.67) less likely to complete the six months follow-up. Including only responders, at six months post cessation, in the multivariate analysis, women were 48% (OR=1.48; 95% CI: 1.04–2.10) more likely compared with men, while snus users were 29% (OR=0.71; 95% CI: 0.45–1.12) less likely compared with non-snus users, to achieve successful smoking cessation.

## DISCUSSION

Our study shows that current snus use was significantly associated with unsuccessful smoking cessation for both men and women. The study is, to the best of our knowledge, the first where the association between current snus use and smoking cessation is based on a large population-based study with smokers, followed for six months after the cessation date. Overall, being a student, and having a medium or high nicotine dependence score were also predictors of unsuccessful smoking cessation. Being female and longer education were predictors of successful smoking cessation.

A recent systematic Swedish review examined the associations between the use of snus (moist tobacco) and tobacco smoking. The review included observational studies with a minimum of 3 months of follow-up; or randomized control trials; including general populations samples, allowing for the comparison between self-reported users and non-users of snus. Only eight studies on snus use and changes (non-use, initiation, decrease, increase, quitting) in the use of combustible tobacco products at follow-up, were identified from 1998 to mid November 2019. None of these studies examined the association between snus use and quitting smoking for at least 30 days, or with decreased smoking^11^. Our study fits the inclusion criteria which were used for examining snus use and subsequently quitting tobacco smoking. Additionally, our results are based on smoking cessation after six months rather than after one month. The result from our follow-up study adds information on predictors of smoking cessation that is impossible to get from cross-sectional studies. Our results add knowledge of a possible cause and effect association between snus use and unsuccessful smoking cessation.

In Norway, the snus prevalence has been increasing and the smoking prevalence has been decreasing during the same calendar time^4^. The two previously mentioned reports suggest that this correlation could be a cause-and-effect association^5,6^. A closer look at the figures in the report shows that this is not the case^4^, but it is beyond the scope of this article to discuss this in detail.

Since the 1990s, new and changed snus products have been introduced in Norway. The snus is produced in tiny pouches, so it is less messy, and the characteristic upper lip protrusion is not as noticeable as when the snus user had to place a pinch of the loose snus tobacco under the upper lip. The pouches were packed in elegant and colorful tin boxes^12^. Flavors of cherry, bubble gum, and mint, were added to make snus more palatable^12,13^.

We think that many of these changes were designed to tap into the young, female market. We do not know if these young women would have remained tobacco-free if snus was not available or whether these women will later initiate tobacco smoking. Lund et al.^14^ reported that as many as 24% of male dual cigarette and snus users, stated snus to be their first tobacco product.

The sale of snus is prohibited in the European Union^15^. Sweden is exempt from this, as they have manufactured and used snus for many decades. Snus has also been a legal consumer product in Norway. A recent ruling in the European Court of Justice upheld the ban on snus in the European Union^16^. Our findings that snus use may be a predictor of unsuccessful smoking cessation, is an additional reason to uphold this ruling.

In our study, students were less likely to successfully quit compared with full-time workers. In an extensive review on nicotine addiction, Prochaska and Benowitz^17^ argued that students are more likely to be dual (tobacco and e-cigarette) users compared with adults and that this may hinder cessation among students. Older age was one of the predictors of successful cessation in a large French population-based study^18^, as well as in a few others^19,20^, but not in the review by Vangeli et al.^21^.

In our study, a high nicotine dependence score was a predictor for unsuccessful smoking cessation, for both snus users and non-users. A report from 2021, describes lower levels of nicotine dependence as the predictor of successful smoking cessation for all ages. However, this large study, including close to 16000 smokers from Canada, the United States, the United Kingdom, and Australia, used only 30 days smoking abstinence as an outcome^22^. Low levels of nicotine dependence have been associated with successful smoking cessation in several studies^19-21,23^.

We found that the female sex and longer education, were the only predictors of successful smoking cessation. However, two reviews, one from 2011^21^ and another from 2016^24^, found large variations in sex as a predictor of smoking cessation.

Longer education as a predictor of successful cessation is in accordance with several longitudinal studies^19,20,25^ and also with several studies collecting quitting information retrospectively^7,18,26^. Two of the latter studies from the European Union Member States used data from the Eurobarometer obtained by face-to-face interviews^7,26^. Considered together, these findings suggest that there will be increasing inequalities in health in the European countries due to differences in successful smoking cessation.

### Strengths and limitations

Our study has several major strengths. It is one of few internet-based intervention studies, that examine longitudinally, six months post cessation, the association between predictors for smoking cessation. In addition, we have a relatively high quit rate at six months post cessation. The study is population-based and included more than 4000 smokers who enrolled to quit smoking. We were able to compare sex, current snus use with non-use, as well as other factors as possible predictors for smoking cessation. We also consider it a strength that e-cigarettes were not available as a consumer product and most likely did not confound our results.

The main limitation of our study is that although we had many smokers enrolled, many participants were lost to follow-up at six months, and the numbers became small for sub-analyses. As noted by Eysenbach^27^, low participation and high dropout rates may be natural and typical features of internet-based eHealth interventions and likely should not be looked upon as failures. When we included only responders in the analyses, the difference between women and men increased, while it decreased for snus users versus non-snus users. In our main analyses, we treat all non-responders as smokers. One reason that we think this is the most likely scenario, is that our intervention study already had a relative high rate of reported successful quitters. If there were many additional quitters among those lost to follow-up, the intervention would have been even more successful. We cannot rule this out, but we think it is unlikely.

Another limitation is that successful smoking cessation is self-reported and not validated by biochemical tests. However, validation studies in Norway and Finland have shown a high degree of concordance between self-reported smoking and biological markers of smoking^28,29^. Thus, there is little reason to believe that the information bias from self-reported smoking has had any substantial effect on the results presented. We experienced a technical error related to the age variable at enrolment, which was discovered and corrected. However, this resulted in difficulties to stratify by age groups, due to missing values. For the other variables, we had less than 5% missing values^8^.

## CONCLUSIONS

Our study identifies female sex and longer education as predictors for successful, while a medium Fagerström score, being a student, and current snus use, were predictors for unsuccessful smoking cessation. Only current snus use was a predictor for unsuccessful quitting for both men and women. Our results indicate that smokers should be warned that snus use may prevent successful smoking cessation.

## Data Availability

The data supporting this research are available from the authors on reasonable request.

## References

[1] Jha P (2013). The 21st century benefits of smoking cessation in Europe. Eur J Epidemiol.

[2] World Health Organization (2020). World Cancer Report: Cancer Research for Cancer Prevention.

[3] Kjønstad A (2000). Tobakksindustriens erstatningsansvar [Tobacco industry liability]: utredning fra en faggruppe med mandat fra Sosial- og helsedepartementet 23. januar 1998: avgitt 28. juni 2000.

[4] (2021). Tobacco, alcohol and other drugs.

[5] Lund KE, Scheffels J, McNeill A (2011). The association between use of snus and quit rates for smoking: results from seven Norwegian cross-sectional studies. Addiction.

[6] Clarke E, Thompson K, Weaver S, Thompson J, O'Connell G (2019). Snus: a compelling harm reduction alternative to cigarettes. Harm Reduct J.

[7] Cheung CM, Vardavas CI, Filippidis FT (2021). Factors associated with abstinence after a recent smoking cessation attempt across 28 European Union member states. Tob Prev Cessat.

[8] Gram IT, Larbi D, Wangberg SC (2019). Comparing the Efficacy of an Identical, Tailored Smoking Cessation Intervention Delivered by Mobile Text Messaging Versus Email: Randomized Controlled Trial. JMIR Mhealth Uhealth.

[9] Wangberg SC, Nilsen O, Antypas K, Gram IT (2011). Effect of tailoring in an internet-based intervention for smoking cessation: randomized controlled trial. J Med Internet Res.

[10] Etter JF, Duc TV, Perneger TV (1999). Validity of the Fagerström test for nicotine dependence and of the Heaviness of Smoking Index among relatively light smokers. Addiction.

[11] Swedish Agency for Health Technology Assessment and Assessment of Social Services (2020). Associations between the use of snus (moist tobacco) or electronic cigarettes and tobacco smoking: A systematic review.

[12] Lund I, Lund KE (2014). How has the availability of snus influenced cigarette smoking in Norway?. Int J Environ Res Public Health.

[13] European Commission (2008). Scientific Committee on Emerging and Newly Identified Health Risks.

[14] Lund KE, McNeill A (2013). Patterns of dual use of snus and cigarettes in a mature snus market. Nicotine Tob Res.

[15] European Commission (2014). Directive 2014/40/EU of the European Parliament and of the Council of 3 April 2014 on the approximation of the laws, regulations and administrative provisions of the Member States concerning the manufacture, presentation and sale of tobacco and related products and repealing Directive 2001/37/EC Text with EEA relevance. OJ L.

[16] Court of Justice of the European Union JUDGEMENT OF THE COURT (First Chamber); 22 November 2018: Reference for a preliminary ruling — Approximation of laws — Manufacture, presentation and sale of tobacco products — Directive 2014/40/EU — Article 1(c) and Article 17 — Prohibition on the placing on the market of tobacco products for oral use — Validity.

[17] Prochaska JJ, Benowitz NL (2019). Current advances in research in treatment and recovery: Nicotine addiction. Sci Adv.

[18] El-Khoury Lesueur F, Bolze C, Melchior M (2018). Factors associated with successful vs. unsuccessful smoking cessation: Data from a nationally representative study. Addict Behav.

[19] Holm M, Schiöler L, Andersson E (2017). Predictors of smoking cessation: A longitudinal study in a large cohort of smokers. Respir Med.

[20] Osler M, Prescott E (1998). Psychosocial, behavioural, and health determinants of successful smoking cessation: a longitudinal study of Danish adults. Tob Control.

[21] Vangeli E, Stapleton J, Smit ES, Borland R, West R (2011). Predictors of attempts to stop smoking and their success in adult general population samples: a systematic review. Addiction.

[22] Arancini L, Borland R, Le Grande M (2021). Age as a predictor of quit attempts and quit success in smoking cessation: findings from the International Tobacco Control Four-Country survey (2002-14). Addiction.

[23] Osler M, Prescott E, Godtfredsen N, Hein HO, Schnohr P (1999). Gender and determinants of smoking cessation: a longitudinal study. Prev Med.

[24] Smith PH, Bessette AJ, Weinberger AH, Sheffer CE, McKee SA (2016). Sex/gender differences in smoking cessation: A review. Prev Med.

[25] Janson C, Künzli N, de Marco R (2006). Changes in active and passive smoking in the European Community Respiratory Health Survey. Eur Respir J.

[26] Bosdriesz JR, Willemsen MC, Stronks K, Kunst AE (2015). Socioeconomic inequalities in smoking cessation in 11 European countries from 1987 to 2012. J Epidemiol Community Health.

[27] Eysenbach G (2005). The law of attrition. J Med Internet Res.

[28] Foss OP, Haug K, Hesla PE, Lund-Larsen PG, Vasli LR (1998). Kan vi stole på egenerklaerte røykevaner?. Tidsskr Nor Laegeforen.

[29] Vartiainen E, Seppälä T, Lillsunde P, Puska P (2002). Validation of self reported smoking by serum cotinine measurement in a community-based study. J Epidemiol Community Health.

